# Deep Neck Infections: The Effectiveness of Therapeutic Management and Bacteriological Profile

**DOI:** 10.3390/medicina61010129

**Published:** 2025-01-15

**Authors:** Geanina Bandol, Mihail Dan Cobzeanu, Mihaela Moscalu, Octavian Dragos Palade, Liliana Moisii, Florentina Severin, Emilia Patrascanu, Florin Mocanu, Andrei Ionut Roman, Bogdan Mihail Cobzeanu

**Affiliations:** 1 Ears Nose Throat (ENT) Department, Grigore T. Popa University of Medicine and Pharmacy, 700115 Iaşi, Romania; geanina-bandol@umfiasi.ro (G.B.); octavian.palade@umfiasi.ro (O.D.P.); liliana.moisii@umfiasi.ro (L.M.); florentina-s-severin@umfiasi.ro (F.S.); 2 Surgery Department, “Grigore T. Popa” University of Medicine and Pharmacy, 700115 Iaşi, Romania; florin.mocanu@email.umfiasi.ro (F.M.); roman.ionut-andrei@umfiasi.ro (A.I.R.); bogdan-mihail.cobzeanu@umfiasi.ro (B.M.C.); 3 Department of Preventive Medicine and Interdisciplinarity, Grigore T. Popa University of Medicine and Pharmacy, 700115 Iaşi, Romania; 4 Department of Anesthesia and Intensive Care, University of Medicine and Pharmacy Grigore T. Popa Iasi, 700115 Iaşi, Romania; patrascanu.emilia@umfiasi.ro

**Keywords:** deep neck infections, upper airway obstruction, length of hospital stay, pathogens, positive bacterial culture

## Abstract

*Background and Objectives*: Deep neck infections (DNIs) are severe diagnoses that can cause serious complications. However, there are insufficient data to predict the evolution of this pathology. This study aims to review the microbiology of DNIs and to identify the factors that influence prolonged hospitalization. *Materials and Methods*: The present retrospective cohort observational analytical study analyzed 138 patients with DNIs who were diagnosed and received surgical treatment over a 8-year period. *Results*: Reduced lymphocyte percentages and increased neutrophil-to-lymphocyte ratios (NLRs) were significantly associated with complications (*p* < 0.001 and *p* = 0.0041, respectively). Laryngotracheal infections were significantly associated with complications (25.53%) (*p* = 0.0004). Diabetes mellitus (DM) and immunocompromised status were strongly associated with complications (*p* < 0.001 and *p* = 0.0056, respectively), establishing these conditions as significant risk factors. Patients with complications experienced substantially longer hospitalizations, with a mean duration of 24.9 days compared to 8.32 days in patients without complications (*p* < 0.001). Complications were observed in 47 patients (34.06%). The most common complications were airway obstruction, which occurred in 26 patients (18.84%), and mediastinitis, which was noted in 31 patients (22.46%). Patients requiring tracheotomy due to airway obstruction had 6.51 times higher odds of long-term hospitalization compared to those without airway obstruction (OR = 6.51; *p* < 0.001). Mediastinitis was associated with a 4.81-fold increase in the odds of prolonged hospitalization (OR = 4.81; *p* < 0.001). Monomicrobial infections were observed in 35.5% of cases, with no significant difference between the short-term (<2 weeks, 37.33%) and long-term (≥2 weeks, 33.33%) hospitalization groups (*p* = 0.8472). Conversely, polymicrobial infections were significantly associated with prolonged hospitalization, occurring in 20.63% of the long-term cases compared to 6.66% of the short-term cases (*p* < 0.001). The most common aerobic bacteria observed were *Staphylococcus aureus* (14.28%), *Streptococcus constellatus* (12.69%) and *Streptococcus viridans* (7.93%) during long-term hospitalization. Comparative analysis of the Kaplan–Meier survival curves based on the presence of infection revealed a significantly lower survival in cases with a positive culture. *Conclusions*: Deep neck infection has a complex pathology, whose therapeutic management remains a challenge in order to reduce the length of hospitalization and mortality.

## 1. Introduction

Cervico-mediastinal suppurations are severe infections with a starting point originating in the otolaryngology (ENT—Ear, Nose, Throat) sphere, in which the fascial spaces of the neck are affected by some collections (abscesses) or by the presence only of inflammation (cellulite) [1]. The complex anatomy of the neck, with the various cervical aponeurosis that delimit the cellulose-fatty and visceral spaces, plays an important role in limiting and spreading the infectious process [2].

Commensal bacteria of the oropharyngeal flora are involved in the etiopathogenesis of cervical suppuration. When a bacterial or viral infection causes a physiological imbalance, these commensal bacteria become aggressive and invasive [3].

Depending on the virulence of the germs, but also on patient’s comorbidities (immunocompromised, cancer, diabetes mellitus (DM), elderly) and treatment with nonsteroidal anti-inflammatory drugs (NSAIDs) or corticosteroids, the infection may remain localized for a long time and then turn into an abscess or progress to a diffuse and extensive infection, sometimes necrotic in the form of necrotic extensive cervical cellulitis or adenophlegmon [4,5].

Deep pharyngeal infections must be treated quickly and appropriately with, effective antibiotic medication and timely surgery. In adults with deep neck infections (DNIs), the consequences are so severe that immediate surgical drainage is required [6].

The systemic disease thought to be most commonly associated with DNIs is DM. While *Klebsiella* is more typical in diabetics, *Steptococci* are the most frequently isolated pathogens in the non-diabetic group [7].

The treatment of this medical-surgical emergency must be commenced early with broad-spectrum antibiotic therapy until the type and sensitivities of the germs from the purulent collection are identified and the surgery is performed. To effectively administer antimicrobial agents to a patient, microbiologic data on the abscess must be obtained [8].

Adequate antimicrobial coverage, surgical drainage and appropriate management of complications remain the cornerstone of treatment for DNIs. Although culture-guided antimicrobial therapy is advocated, empirical antibiotic treatment is important before the culture results become available. The administration of empirical antibiotics plays a critical role in alleviating the clinical course of the disease [9]. DNIs have the potential to induce serious complications.

The rapid onset and progression of DNIs and their potential for high morbidity and mortality rates can impose significant physical, psychological and economic burdens on affected patients and their families [6]. The literature suggests that the hospitalization duration for DNIs is generally about 2 weeks [5,6]. However, the exact duration depends on the complications that occur and whether the infection involves multiple neck spaces, although these findings do not show a general consensus [5,7,8]. Global mortality rates from DNIs vary from 0 to 2.7% [4,9], with death being associated with the presence of significant comorbidities. However, few studies present predictive models for DNI mortality or complications explored prior to treatment.

Considering the findings presented so far in the specialized literature [8,9], new assessments of the risk factors and bacteriological profile of surgically treated DNI patients are required.

The aim of this study is to review the bacteriology of DNIs and to identify their impact on the effectiveness of therapeutic management. 

This should make it possible to update the bacteriological data and thus guide practitioners in the choice of probabilistic antibiotics. Deep neck infections have the potential to induce serious complications. This study was designed so that, based on the results obtained, the evolution of patients with this pathology could be evaluated.

Our study creates a bacteriological profile of these patients from the point of view of the effectiveness of the therapy. The effectiveness of the therapy was evaluated by the complications that occurred and the length of stay in hospital of these patients.

The research question of this study is what are the factors that significantly influence the occurrence of complications in patients with deep throat infections treated surgically. Another question was which of the complications that occurred significantly influence the duration of hospitalization and what is the bacteriological profile of the patients who require a long hospitalization.

## 2. Materials and Methods

### 2.1. Study Group: Inclusion and Exclusion Criteria

We performed a retrospective cohort observational analytical study over a period of 8 years (from 1 January 2016 to 31 December 2023) of DNIs in patients treated in the otolaryngology and oromaxilofacial departments of the University Hospital “St. Spiridon” in Iasi, Romania.

The protocol of the study was approved by the local ethical review board (No. 15/01.03/2021). The written consent of all patients included in the study was obtained. In this retrospective analytical-observational cohort study, the medical data recorded in the patients’ electronic records were used.

The inclusion criteria included hospitalized patients diagnosed with deep cervical suppuration who had aspiration of purulent secretions based on bacteriological examination and CT examination and who required surgery. The exclusion criteria included patients with fistulized or incised peritonsillar phlegmon not affecting the deep cervical spaces, patients with superficial infections in the cervical region that did not require surgical interventions, and patients with fistulized intra-oral abscesses.

Bacteriological culture and sensitivity tests were performed using pus cultures or throat swabs. In all cases, the patients underwent a surgical procedure to drain the pus. The diagnosis of DNIs was suspected on the basis of clinical history and confirmed by computed tomography (CT) or surgery. This analysis excluded patients with cervical infections who did not require surgery, such as cellulitis, and superficial or limited infections.

**Figure 1:**
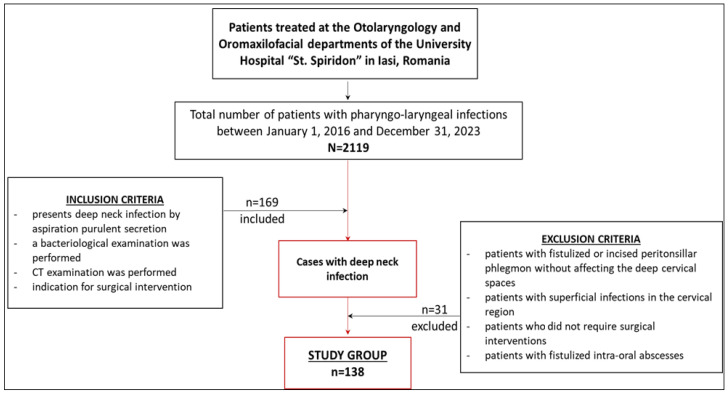
Study flow chart—study group selection.

Of the total of 2119 cases presented in the clinic with pharyngo-laryngeal infections, after applying the inclusion and exclusion criteria, only 169 patients were eligible for inclusion in the study. Of these, we also excluded 14 cases with hematoma or thoracic abscesses with deep neck involvement and 17 cases with neck hematomas whose data were incomplete. The group of patients that was studied included 138 patients with deep neck abscesses that required surgical intervention (Figure 1).

### 2.2. Study Design

In the present study, the efficacy of the therapeutic management of DNIs was evaluated based on morbidity, mortality and the length of hospitalization. Long-term hospitalization was defined as a hospital stay exceeding 14 days.

The efficacy of the therapeutic management was evaluated based on demographic factors (gender, age), disease-related symptoms, etiology, bacteriology, systemic diseases (comorbidities), microbiological findings and the length of hospitalization.

Inflammatory laboratory indicators such as leukocytes, lymphocytes, neutrophils and C-reactive protein (CRP) were also analyzed. Systemic comorbidities that were assessed included conditions that could influence disease progression, particularly cardiovascular or pulmonary diseases, DM, liver disease, hematological disorders and other severe illnesses.

The anatomical spaces involved were classified according to the existing literature, including the following regions identified in this study: submandibular and retrostylian, parapharyngeal and prevertebral, lateral cervical, anterior cervical, retropharyngeal and mediastinal extension. If two or more spaces were significantly involved simultaneously, these cases were classified as having multispace involvement.

The diagnosis of DNIs was confirmed through CT, needle aspiration, or surgical intervention.

The treatment of DNIs was evaluated retrospectively, covering the period from disease onset to surgical incision. All 138 patients received intravenous antibiotic therapy. The selection of antibiotics was determined based on patient age, comorbidities and whether the patient had previously undergone antibiotic treatment. Antibiotic regimens were subsequently adjusted according to culture results and the patients’ clinical response.

*Microbiology*. Cultures were obtained for all 138 patients to identify both aerobic and anaerobic pathogens. Pathogen identification within the first 24–48 h was performed through bacteriological examination of the abscess secretions, obtained via puncture or from surgical wounds. Pathogen identification was conducted using the MALDI-TOF apparatus, while antibiograms were processed using Team Freedom Evo cards. The monitoring of bacteriological clearance was performed following the implementation of targeted therapy based on the antibiogram results.

*Laboratory procedures and parameters.* An oral examination, an endoscopy of the upper aerodigestive tract, an orthopantography of the mandible and a contrast-enhanced CT neck and thorax were part of the routine examination.

Pus was collected during the surgery and drainage. This was collected for microbiological analysis by aspiration in a syringe of 5–10 mL and 18/22 gauge needle under aseptic conditions. The incubation lasted 48 h. In addition, these patients were not under antibiotic medication before the onset of the infectious pathology. Different antibiotics were tested at the end of this stage, and an antibiogram was performed.

We classified the bacteria into different categories based on the characteristics of Gram staining and anaerobic properties. In our hospital, high-resolution MALDI-TOF MS (matrix-assisted laser desorption and ionization time-of-flight mass spectrometry) was used to identify the majority of gram-positive and gram-negative bacterial strains to species level. Antibiograms were generated on the Freedom EVO platform and interpreted using the EUCAST reading standard.

### 2.3. Statistical Analysis

For the statistical analysis, SPSS v.29 (IBM Ireland Product Distribution Limited, IBM House, Shelbourne Road, Ballsbridge, Dublin 4, Ireland) was used. Descriptive statistics included the mean with standard deviation (SD), median with 25–75th percentiles (interquartile range (IQR)) and absolute (n) with relative (%) frequencies. The comparisons between the analyzed groups were performed using Student’s *t*-test or Mann–Whitney U Test for continuous variables, depending on the homogeneity of the data series, based on Levene’s test. The qualitative variables were presented as absolute (n) and relative (%) frequencies, and the comparison among the groups was based on the results of the Pearson Chi-square test. Univariate logistic regression analyzed the effect level (complications) by the odds ratio (OR) and its confidence interval (CI). Survival analysis was performed using the Kaplan–Meier methodology to assess overall survival, defined as the interval between the date of hospitalization and the estimated date of death in days. Statistical significance was set at *p* < 0.05.

## 3. Results

### 3.1. Patient Characteristics

The retrospective study was conducted on a cohort of 138 patients with DNIs, comprising 108 males (78.26%) and 30 females (21.74%). The mean age of the analyzed group was 52.9 years, with a standard deviation of 14.9. In the initial phase of the study, the patients were divided into two groups based on the presence of complications. All the clinical and demographic aspects were comparatively analyzed, with the presence of complications serving as the primary outcome of interest (Table 1).

The analysis revealed no significant difference in the male-to-female ratio between patients with and without complications (*p* = 0.2315), suggesting an absence of gender-specific predisposition to complications. However, the patients experiencing complications were significantly older, with a mean age of 59.2 years compared to 49.8 years in those without complications (*p* = 0.0003), identifying age as a potential risk factor (Table 1).

Elevated leukocyte counts and neutrophil ratios were observed in patients with complications (*p* = 0.0235 and *p* < 0.001, respectively), indicating a heightened inflammatory response. In addition, reduced lymphocyte percentages and increased neutrophil-to-lymphocyte ratios (NLRs) were significantly associated with complications (*p* < 0.001 and *p* = 0.0041, respectively), underscoring their relevance as indicators of disease severity. Markedly elevated ESR (*p* = 0.0128) and fibrinogen levels (*p* < 0.001) further suggest a systemic inflammatory state (Table 1).

Patients with complications demonstrated significantly higher creatinine and presepsin levels (*p* = 0.0017 and *p* = 0.0278, respectively), reflecting potential renal impairment and sepsis progression. Elevated CRP and blood glucose levels (*p* < 0.001 for both) were also observed, indicating systemic inflammation and possible metabolic disturbances, such as stress-induced hyperglycemia (Table 1).

The involvement of the lateral cervical space was not associated with the presence of complications; the analysis results indicated a significantly lower frequency of complications for this location (55.31% vs. 60.43%; *p* = 0.0261). In contrast, for retropharyngeal infections, the frequency of complications was significantly higher (19.14% vs. 8.79%; *p* = 0.0046). A particularly strong association was identified between cervical mediastinitis and complications (*p* = 0.0005), emphasizing its role as a critical determinant of disease severity. Furthermore, infections spanning multiple anatomical spaces were significantly more prevalent among the patients with complications (*p* = 0.0305), indicating that the extent of spread correlates with increased severity.

From an etiological perspective, laryngotracheal infections were significantly associated with an increased frequency of complications, with 25.53% of complications having laryngotracheal infection as the etiological factor (*p* = 0.0004). In contrast, for congenital cysts or trauma, the frequency of complications was significantly lower (*p* = 0.0351), with only 4.25% of complications attributed to this etiological factor.

Symptoms including pain, dysphagia and dyspnea were also notably more common in the patients with complications (*p* = 0.0032, *p* = 0.0018 and *p* = 0.0009, respectively). Additional symptoms, such as chest pain and dysphonia, demonstrated significant associations as well (*p* = 0.0014 and *p* = 0.0016, respectively).

Comorbidities, particularly DM and immunocompromised status, were strongly associated with complications (*p* < 0.001 and *p* = 0.0056, respectively), establishing these conditions as significant risk factors. Furthermore, alcohol abuse was significantly more frequent in the complication group (*p* = 0.0140).

The patients with complications experienced substantially longer hospitalizations, with a mean duration of 24.9 days compared to 8.32 days for the patients without complications (*p* < 0.001). Mortality was significantly higher among the patients with complications, at 25.53%, compared to 0% in those without complications (*p* < 0.001), highlighting the life-threatening nature of severe cases.

### 3.2. Duration of Hospitalization

The efficacy of therapeutic management is clearly reflected in the length of hospitalization. To provide a comprehensive analysis of the therapeutic management of patients with DNIs, we examined the impact of complications on the duration of hospital stays.

**Figure 2:**
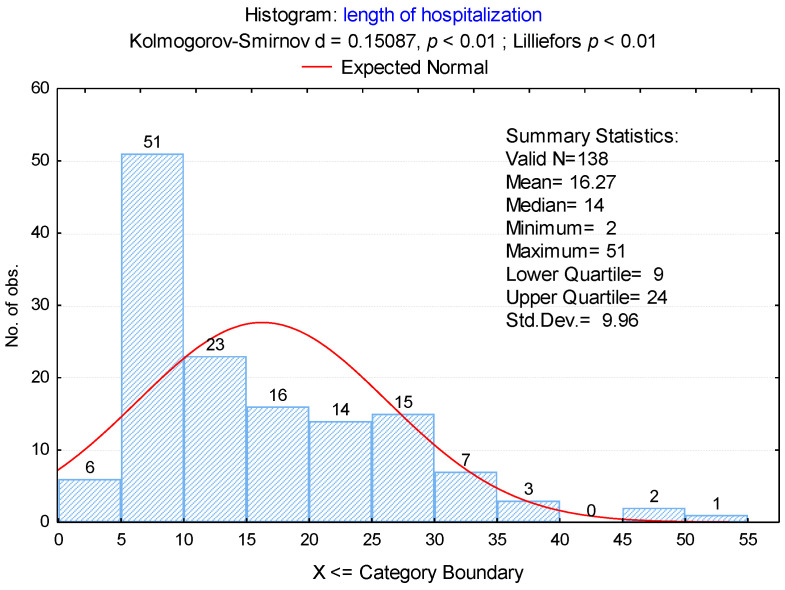
Duration of hospitalization in patients with DNIs.

In this study, the patients were divided into two groups based on their hospitalization duration: those with stays shorter than 2 weeks and those with stays longer than 2 weeks. The average length of hospitalization was 16.3 days (standard deviation: 9.9 days), with a range between 2 and 51 days (Figure 2).

### 3.3. Complications and Their Association with the Duration of Hospitalization

Complications were observed in 47 patients (34.06%). The most common complications were airway obstruction, which occurred in 26 patients (18.84%), and mediastinitis, which was noted in 31 patients (22.46%) (Table 2).

The cases with mediastinitis that required a hospital stay of less than 14 days were cases with damage to the superior mediastinum.

The statistical analysis examines the association between complications and long-term hospitalization (over 2 weeks) in patients with DNIs (Table 2). A univariate logistic regression model was used to estimate the odds ratio (OR), confidence intervals (CIs) and statistical significance (*p*-value) for each complication.

Patients requiring a tracheotomy due to airway obstruction had 6.51 times higher odds of long-term hospitalization compared to those without airway obstruction (OR = 6.51; 95% CI: 2.27–18.62; *p* < 0.001). This highlights airway obstruction as a critical complication significantly associated with prolonged hospital stays. Internal jugular vein thrombosis was associated with a 5.01-fold increase in the likelihood of extended hospitalization (OR = 5.01; 95% CI: 3.54–11.09; *p* = 0.0154). Although less frequent (3.62%), this condition substantially impacts morbidity and hospitalization duration. Patients with pneumonia had 3.49 times higher odds of long-term hospitalization (OR = 3.49; 95% CI: 2.89–9.78; *p* = 0.0174). Pneumonia, as a secondary complication, further exacerbates recovery time in patients with DNIs. Mediastinitis was associated with a 4.81-fold increase in the odds of prolonged hospitalization (OR = 4.81; 95% CI: 2.96–9.72; *p* < 0.001). As a severe and life-threatening complication, mediastinitis significantly influences the prognosis and resource utilization. Patients with necrotizing fasciitis were 2.26 times more likely to experience long-term hospitalization (OR = 2.26; 95% CI: 1.23–6.17; *p* = 0.027). Although less common (4.35%), its association with prolonged stays reflects its severity and need for extensive treatment (Table 2).

Conversely, spontaneous fistulization was not significantly associated with long-term hospitalization (OR = 1.83; 95% CI: 0.65–5.14; *p* = 0.2491). This suggests that while spontaneous fistulization occurs in certain cases, it does not independently contribute to the duration of hospital stay. Similarly, renal insufficiency showed no statistically significant association with prolonged hospitalization (OR = 1.39; 95% CI: 0.55–3.86; *p* = 0.5210). Despite its occurrence in 12.31% of the patients, renal insufficiency did not appear to be a decisive factor in determining hospitalization duration (Table 2).

### 3.4. Positive Bacterial Culture Associated with Long-Term Hospitalization

All 138 patients initially received broad-spectrum intravenous antibiotics, with the therapy subsequently adjusted based on the culture results and the antibiotic sensitivity report. A positive culture was obtained from 70 patients (50.72%).

Monomicrobial infections were observed in 35.5% of cases, with no significant difference between the short-term (<2 weeks, 37.33%) and long-term (≥2 weeks, 33.33%) hospitalization groups (*p* = 0.8472). Conversely, polymicrobial infections were significantly associated with prolonged hospitalization, occurring in 20.63% of the long-term cases compared to 6.66% of the short-term cases (*p* < 0.001). Specifically, infections involving two pathogens (12.69%) or three pathogens (7.93%) were notably more frequent in the patients requiring long-term hospitalization compared to the short-term cases (5.33% and 1.33%, respectively; *p* < 0.001 for both). Polymicrobial infections are a strong predictor of extended hospital stays, suggesting increased clinical complexity and severity in these cases (Table 3).

Several gram-positive aerobic species demonstrated significant associations with prolonged hospitalization. For example, *Streptococcus viridans*, was observed in 7.93% of the long-term hospitalization cases compared to 5.33% of the short-term hospitalization cases (*p* = 0.0354) and *Staphylococcus aureus* was observed in 14.28% of the long-term hospitalizations versus 10.66% of the short-term hospitalizations (*p* = 0.0361). Group C Streptococci occurred in 4.76% of the long-term stays compared to 6.66% of the short-term cases (*p* = 0.0247) and *B-hemolytic streptococci* was significantly more common in the long-term hospitalizations (6.34%) compared to the short-term cases (2.66%, *p* = 0.0019). *Streptococcus constellatus* was present in 12.69% of the long-term hospitalizations versus 8.00% of the short-term cases (*p* = 0.0286) (Table 3).

Pseudomonas aeruginosa and *Klebsiella pneumoniae* were also significantly associated with prolonged hospital stays (*p* < 0.001 for both). While pathogens such as *Escherichia coli* (*p* = 0.1558) and *Haemophilus influenzae* (*p* = 0.6882) did not exhibit significant associations, Acinetobacter species were significantly more prevalent among the patients with prolonged hospitalization (6.34%) compared to the short-term cases (2.66%, *p* = 0.0311) (Table 3).

Among the gram-negative aerobes, *Acinetobacter species* emerged as a significant contributor to extended hospital stays, reflecting its clinical relevance in severe infections. Notably, no microbial growth was reported in 62.66% of the short-term hospitalization cases, whereas no such instances were noted among the long-term hospitalizations (*p* < 0.001) (Table 3).

### 3.5. Mortality

**Figure 3:**
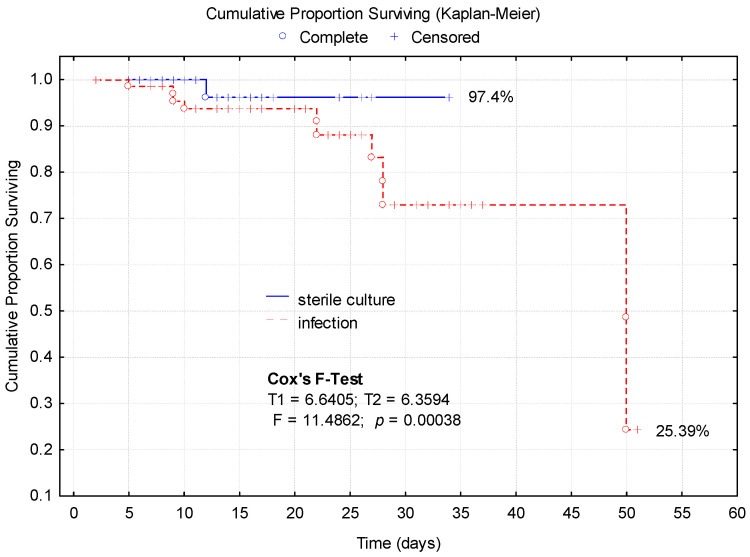
Kaplan–Meier survival curves according to the presence of infection.

The comparative analysis of the Kaplan–Meier survival curves according to the presence of infection revealed a significantly lower survival in cases with a positive culture (Figure 3).

The patients with sterile cultures exhibited higher survival probabilities, with a final survival rate of 97.4%. The patients with positive infections demonstrated significantly worse outcomes, with survival dropping to 25.39% (Figure 3).

During the first 22 days of hospitalization, the two groups of patients (with infection/sterile culture) showed a survival rate without significant differences. After this interval, the probability of survival for the group with infections dropped sharply, reaching 72% after 27 days of hospitalization. The sterile culture group maintained a relatively flat curve, indicating a minimal event rate (mortality). Cox’s F-test provides a measure of the difference between the two groups (F = 11.48, *p* = 0.00038) (Figure 3).

## 4. Discussion

The present study evaluates the therapeutic management and bacteriological profile of patients with DNIs, focusing on morbidity, complications and their association with prolonged hospitalization and mortality. The literature has reported an incidence of DM of 16% to 20% [10]. Arterial hypertension, which can be associated with cardiac and pulmonary diseases, is not given importance, but these factors can have an influence on morbidity and mortality in DNIs [11,12].

The findings contribute significantly to understanding the predictors of disease severity, particularly in the context of bacteriological characteristics and systemic inflammatory responses [13,14].

The analysis highlights key risk factors and markers associated with complications in DNIs. Older age, systemic inflammatory markers (NLRs, ESR, CRP), multispace involvement and comorbidities like diabetes are significant predictors [11]. Complications correlate with prolonged hospitalizations and increased mortality, emphasizing the need for early therapeutic intervention (antibiotic and surgical treatment) and monitoring of high-risk patients.

In our study, complications such as airway obstruction, internal jugular vein thrombosis, pneumonia, mediastinitis and necrotizing fasciitis significantly increased the odds of prolonged hospitalization in patients with DNIs. Airway obstruction and mediastinitis had the strongest associations with long-term hospitalization, reflecting their critical impact on patient outcomes. The nonsignificant results obtained in this study for spontaneous fistulization and renal failure suggest that these factors may not independently influence the length of hospital stay.

These findings emphasize the need for early detection and effective management of severe complications to reduce morbidity and hospital stays in patients with DNIs. Preventative strategies for complications like pneumonia and necrotizing fasciitis, including close monitoring and timely intervention, can help mitigate the risk of prolonged hospital stays.

Mediastinitis is a fatal infectious disease in which a DNI spreads along the cervical fascia and neck spaces and down to the mediastinum [15,16,17]. The spread of an infection to the mediastinum is facilitated by gravity and negative intrathoracic pressure during breathing [18,19]. The most important route for a descending infection reported in the literature is the retropharyngeal space [20,21]. Celakovsky et al. reported the predisposing factors for mediastinal extension of a DNI as cardiovascular and pulmonary diseases [22]. Some studies have shown that risk factors such as DM, chronic renal failure and alcoholism contribute to the extension of DNIs into the mediastinal space [23,24].

Tapiovaara et al. also found that mediastinal involvement prolongs the need for hospitalization [25]. Currently, multidisciplinary approaches and comprehensive medical treatment can significantly reduce the mortality caused by mediastinitis [26]. The DNIs are usually polymicrobial in nature [27,28]. The most common cause of DNIs is considered to be odontogenic, and the oral flora are composed of a mixture of aerobic and anaerobic bacteria [29,30].

Complications were observed in 34.06% of the patients, with airway obstruction (18.84%) and mediastinitis (22.46%) being the most frequent. These complications significantly influenced the duration of hospitalization, with mediastinitis increasing the likelihood of prolonged stays (OR = 4.81, *p* < 0.001). This aligns with prior research demonstrating that mediastinitis remains a life-threatening condition associated with increased morbidity and mortality, particularly in cases involving the superior mediastinum [31].

Airway obstruction requiring a tracheotomy further highlighted its role as a critical determinant of prolonged hospitalization (OR = 6.51, *p* < 0.001). These findings reflect previous studies emphasizing the need for timely airway management to prevent hypoxia and other adverse outcomes [32,33].

Conversely, spontaneous fistulization and renal insufficiency were not significantly associated with extended hospitalization, suggesting that these conditions may be less severe or effectively managed through appropriate interventions. However, the presence of comorbidities such as DM (55.31% in the complication group) and immunosuppression (34%) significantly influenced outcomes. Diabetes, in particular, predisposes patients to infections by impairing neutrophil function and tissue perfusion, consistent with findings from Bal K.K. and Boscolo-Rizzo P. [3].

Previous studies show that the most common gram-positive strain was *Streptococcus* and the most common gram-negative strain was *Klebsiela pneumoniae*. Interestingly, there are studies that claim that only DM was mainly associated with *Klebsiela pneumoniae* [34,35].

Therefore, third-generation cephalosporins, piperacillin and quinolones can be recommended as empiric antibiotics to treat patients with deep neck infections but mild forms (gram-negative strains), while penicillin and first- or second-generation cephalosporins can be recommended as an empiric antibiotic in patients with mild forms of DNI without DM (gram-positive strains) [36,37]. The low rates of resistance to these antibiotics also support these findings [38,39].

In our study, polymicrobial infections, particularly those involving multiple pathogens, are significantly associated with prolonged hospitalization, likely due to the increased severity and treatment complexity. Specific pathogens, including *Streptococcus species*, *Staphylococcus aureus*, *Pseudomonas aeruginosa* and *Klebsiella pneumoniae*, play a critical role in determining the hospitalization duration. Among the gram-negative bacteria, *Acinetobacter species* demonstrated a significant association with extended hospital stays. The absence of microbial growth in the short-term cases highlights the importance of timely intervention and the potential limitations of diagnostic methods. Many studies have confirmed that DNIs most commonly involve mixed infections of aerobic and anaerobic strains [40,41,42].

These findings emphasize the need for early identification and targeted management of polymicrobial and pathogen-specific infections to reduce hospitalization duration and improve patient outcomes. The International Guidelines for the Management of Sepsis (2016) mention indications in the case of poorly controlled infection with the risk of sepsis from an unidentified pathogen [43].

The study revealed that polymicrobial infections were significantly associated with prolonged hospitalization, occurring in 20.63% of the long-term cases compared to 6.66% of the short-term cases (*p* < 0.001). This result supports the hypothesis that infections involving multiple pathogens are more severe and clinically complex, often requiring broader-spectrum antibiotics and longer treatment durations [44].

Among specific pathogens, gram-positive aerobes such as *Streptococcus viridans* (7.93%, *p* = 0.0354) and *Staphylococcus aureus* (14.28%, *p* = 0.0361) were prevalent in prolonged hospitalizations. These findings align with the current literature, which identifies Streptococcus species as primary causative agents in DNIs, with *Staphylococcus aureus* contributing significantly to abscess formation and disease progression [45].

Gram-negative pathogens such as *Pseudomonas aeruginosa* and *Klebsiella pneumoniae* were also significantly associated with prolonged hospitalization (*p* < 0.001). *Acinetobacter species* emerged as a significant contributor to extended stays (6.34%, *p* = 0.0311), reflecting their role in severe infections, particularly in immunocompromised individuals. These findings highlight the importance of early identification and targeted antimicrobial therapy to prevent prolonged morbidity.

Interestingly, in cases where no microbial growth was detected, the majority of the patients experienced shorter hospitalization durations (62.66% of short-term cases, *p* < 0.001). This could suggest that early empirical antibiotic therapy effectively suppresses bacterial growth, thereby limiting disease progression.

Inflammatory markers such as leukocytes, NLRs and CRP demonstrated significant associations with complications and prolonged hospital stays. Elevated NLRs (*p* = 0.0041) and CRP levels (*p* < 0.001) in the patients with complications corroborate the findings of previous studies that these markers serve as reliable indicators of systemic inflammation and disease severity.

Hyperglycemia, often observed in diabetic and critically ill patients, was also significantly associated with complications and prolonged hospitalization (*p* < 0.001). Stress-induced hyperglycemia has been widely recognized as a predictor of poor outcomes in infections, particularly among immunocompromised individuals.

The Kaplan–Meier survival analysis revealed a significantly lower survival rate in the patients with positive bacterial cultures, with survival probabilities dropping to 25.39% compared to 97.4% in the patients with sterile cultures (*p* = 0.00038). These results underscore the detrimental impact of active infections on patient prognosis, particularly when complicated by sepsis or mediastinitis. The steep decline in survival after 22 days highlights the importance of aggressive early intervention, aligning with previous findings on the role of culture-positive infections in predicting mortality.

This study emphasizes the importance of early diagnosis and intervention, prompt airway management, surgical drainage and initiation of broad-spectrum antibiotics as essential to reduce morbidity. Empirical antibiotic regimens must be reviewed based on culture and sensitivity results to optimize outcomes and reduce hospitalization duration.

Biomarkers such as NLRs and CRP should be integrated into routine clinical assessment to identify patients at high risk of complications. Effective glycemic control and management of underlying systemic diseases are essential in reducing the severity and progression of DNIs.

However, the study also has limitations. A series of variables that present important elements in the evolution of patients with DNIs were not included in the analysis. Therefore, we should look for other causes of this increase in long-term hospitalization, such as the administration of self-medication in patients with DNIs at the time of symptom onset (use of steroid and nonsteroidal anti-inflammatories, antibiotic treatment) as well as the time that elapsed from symptom onset to hospitalization. The lack of oral hygiene in certain groups of patients (smokers, alcohol consumers, patients with poor economic conditions) should not be neglected, as they can increase the frequency of odontogenic infections with the appearance of cervico-mediastinal complications and, implicitly, the duration of hospitalization.

In the various published studies, a protocol has not yet been developed to indicate the timing of tracheotomy. This would reduce the number of complications and mortality through easier access to surgical reinterventions and the decrease in the time spent by the patient in the intensive care unit, which would reduce the number of nosocomial infections.

This study highlights the critical role of complications, such as mediastinitis and airway obstruction, in determining hospitalization duration and mortality among patients with DNIs. The findings emphasize the need for early intervention, targeted therapy and the management of systemic risk factors to improve clinical outcomes. Further research on predictive models integrating clinical, bacteriological and inflammatory markers could enhance the management of this life-threatening condition.

The retrospective study carried out limits the ability to establish causal relationships between the risk factors analyzed and the complications that occurred. To solve this problem, prospective studies are needed to identify predictive factors of the evolution of patients with this pathology. A multivariate analysis of these factors could correctly identify the confounding factors (variables) and thus increase the validity of the conclusions.

## 5. Conclusions

The present analysis highlights key risk factors and markers associated with complications in DNIs. Older age, systemic inflammatory markers (NLRs, ESR, CRP), multispace involvement and comorbidities like diabetes are significant predictors. Also, polymicrobial infections are a strong predictor of extended hospital stays, suggesting increased clinical complexity and severity in these cases.

The presence of specific gram-positive aerobic pathogens, notably *Streptococcus species* and *Staphylococcus aureus*, as well as gram-negative bacteria like *Pseudomonas aeruginosa* and *Klebsiella pneumoniae*, correlates with extended hospitalization durations, underscoring their clinical impact. The absence of detectable microbial growth in a substantial proportion of the short-term cases may indicate effective early treatment or limitations in pathogen isolation techniques. Complications correlate with prolonged hospitalizations and increased mortality, emphasizing the need for early therapeutic intervention (antibiotic and surgical treatment) and monitoring of high-risk patients.

## Figures and Tables

**Table 1:** Baseline characteristics of the patients with DNIs.

Characteristics	Study Group N = 138 Cases	Complications of DNI		*p*-Value *
		Present, n = 47	Absent, n = 91	
Gender, Male/female, n (%)	108/30 (78.26/21.74)	34/13 (31.48/43.33)	74/17 (68.52/56.67)	0.2315
Age, years, mean (SD)	52.9 (14.9)	59.2 (13.9)	49.8 (14.4)	**0.0003**
Smoking	39 (28.26)	35 (74.47)	4 (4.40)	**<0.001**
Alcohol abuse	6 (4.34)	6 (0.12)	1 (0.01)	**0.0140**
**Blood test—Laboratory test**				
Leukocytes, mm^3^, mean (SD)	15418.2 (5161.6)	16164.4 (5250.9)	12032.8 (5101.2)	**0.0235**
Neutrophil ratio (%)	109.2 (10.5)	131.6 (11.5)	98.8 (9.7)	**<0.001**
Lymphocyte (%)	9.4 (6.7)	6.2 (5.3)	10.1 (7.6)	**<0.001**
NLR	12.1 (9.6)	19.8 (8.8)	10.2 (8.4)	**0.0041**
ESR (erythrocyte sedimentation rate), mean (SD)	49.2 (16.75)	56.3 (14.5)	44.7 (11.2)	**0.0128**
Creatinine (μmol/L)	76.9 (11.6)	119.4 (21.3)	61.8 (9.5)	**0.0017**
Fibrinogen, mg/dL, mean (SD)	588.9 (95.6)	639.4 (97.7)	563.3 (84.0)	**<0.001**
Presepsin, pg/mL, mean (SD)	573.5 (454.7)	967.1 (709.1)	295.7 (173.9)	**0.0278**
C-reactive protein, mg/L, mean (SD)	125.8 (22.8)	138.1 (27.5)	119.4 (17.2)	**<0.001**
Blood sugar, mg/dL, mean (SD)	125.8 (58.5)	160.7 (82.2)	103.4 (21.1)	**<0.001**
**Location**				
submandibular and retrostylian	17 (12.31)	6 (12.76)	11 (12.08)	0.1579
parapharyngeal and prevertebral	28 (20.28)	9 (19.14)	19 (20.87)	0.0897
lateral cervical	81 (58.69)	26 (55.31)	55 (60.43)	**0.0261**
anterior cervical	26 (18.84)	8 (17.02)	18 (19.78)	0.0579
retropharyngeal	17 (12.31)	9 (19.14)	8 (8.79)	**0.0046**
mediastini	22 (15.94)	20 (42.55)	2 (2.19)	**0.0005**
**Multispace involvement**	50 (36.23)	28 (56)	22 (44)	**0.0305**
**Etiology factors**				
odontogenic infections	15 (10.86)	5 (10.63)	10 (10.98)	0.1208
pharyngo-tonsillar infections	85 (61.59)	28 (59.57)	57 (62.63)	0.0908
epiglottis	21 (15.21)	10 (21.27)	11 (12.08)	**0.0117**
foreign body	3 (2.17)	1 (2.12)	2 (2.19)	0.0869
congenital cyst or trauma	8 (5.79)	2 (4.25)	6 (6.59)	**0.0351**
laryngotracheal infections	14 (10.14)	12 (25.53)	2 (2.19)	**0.0004**
lymphadenopathy	15 (10.86)	5 (10.63)	10 (10.98)	0.3275
**Symptoms**				
pain	54 (39.13)	15 (31.91)	39 (42.85)	**0.0032**
sore throat	110 (79.71)	36 (76.59)	74 (81.31)	**0.0057**
dysphagia	104 (75.36)	39 (82.97)	65 (71.42)	**0.0018**
dyspnoea	15 (10.86)	14 (29.78)	7 (7.69)	**0.0009**
dysphonia	8 (5.79)	5 (10.63)	3 (3.29)	**0.0016**
otalgia	5 (3.62)	6 (12.76)	3 (3.29)	**0.0056**
chest pain	4 (2.89)	11 (23.4)	5 (5.49)	**0.0014**
fever	69 (50)	23 (48.93)	52 (57.14)	**0.0621**
**Comorbidity, present, n(%)**	**61 (44.20)**	**40 (85.11)**	**21 (23.08)**	**<0.001**
Diabetes mellitus	35 (25.36)	26 (55.31)	9 (9.89)	**<0.001**
Cardiovascular diseases	40 (28.98)	29 (0.61)	11 (0.12)	0.0945
Immunocompromised status	25 (18.11)	16 (0.34)	9 (0.09)	**0.0056**
Malignant tumors	4 (2.89)	4 (0.08)	1 (0.01)	0.0858
Number of days of hospitalization, mean (SD) median (IQR)	16.3 (9.9) 14 (9–34)	24.9 (12.2) 26 (11–45)	8.32 (7.02) 11 (8–17)	**<0.001**
Non-long-term (<2 weeks) Long-term (≥2 weeks)	75 (54.34) 63 (45.65)	13/62 17.33/82.67	34/29 53.97/46.03	**<0.001**
**Mortality**	12 (8.70)	12 (25.53)	0 (0)	**<0.001**

Continuous variables were expressed as median (interquartile range (IQR)); the variables did not have a normal distribution (data not normally distributed); mean (standard deviation (SD)), the variables did have a normal distribution. Categorical variables: number (%). * t Student’s test or Mann–Whitney U Test for continuous variables; Pearson Chi-square test for qualitative variables. Bold *p*-values are statistically significant.

**Table 2:** Association of length of hospital stay (long-term, i.e., more than 2 weeks) with morbidity in patients with DNIs.

Dependent Variable: Long-Term Hospitalization (More Than 2 Weeks)	N = 138 n (%)	Univariate Model:		
Independent Variable: Complications		OR	95%CI for OR	*p*-Value
**Airway Obstruction—Tracheotomy**	26 (18.84)	6.51	2.27–18.62	**<0.001**
**Internal Jugular Vein Thrombosis**	5 (3.62)	5.01	3.54–11.09	**0.0154**
**Pneumonia**	11 (7.97)	3.49	2.89–9.78	**0.0174**
**Mediastinitis**	31 (22.46)	4.81	2.96–9-72	**<0.001**
**Necrotizing fasciitis**	6 (4.35)	2.26	1.23–6.17	**0.027**
**Spontaneous fistulization**	17 (12.31)	1.83	0.65–5.14	0.2491
**Renal insufficiency**	17 (12.31)	1.39	0.55–3.86	0.5210

Logistic regression (Enter—Method); CI, confidence interval; OR, odds ratio. Bold *p*-values are statistically significant.

**Table 3:** The pathogen spectrum in DNIs and the association with the length of hospital stay of patients.

Pathogens	Group Study N = 138 Cases	Length of Hospital Stay		*p*-Value *
		Non-Long-Term (<2 Weeks) n = 75	Long-Term (≥2 Weeks) n = 63	
**Positive bacterial culture**	**70 (50.7)**	**25 (35.7)**	**45 (64.3)**	**<0.001**
**Species**				
Monomicrobial	49 (35.5)	28 (37.33)	21 (33.33)	0.8472
Gram-positive aerobe	39 (28.26)	21 (28)	18 (28.57)	0.9791
Gram-negative aerobe	10 (7.24)	6 (8.00)	4 (6.34)	0.0845
Polymicrobial	18 (13.04)	5 (6.66)	13 (20.63)	**<0.001**
2 pathogens	12 (8.69)	4 (5.33)	8 (12.69)	**<0.001**
3 pathogens	6 (4.34)	1 (1.33)	5 (7.93)	**<0.001**
Gram-positive aerobes				
*Streptococcus viridans*	9 (6.52)	4 (5.33)	5 (7.93)	**0.0354**
*Staphylococcus aureus*	17 (12.31)	8 (10.66)	9 (14.28)	**0.0361**
Group C Streptococci	8 (5.79)	5 (6.66)	3 (4.76)	**0.0247**
*Streptococus B hemolitic*	6 (4.34)	2 (2.66)	4 (6.34)	**0.0019**
*Streptococcus constellatus*	14 (10.14)	6 (8.00)	8 (12.69)	**0.0286**
*Streptococcus anginosus*	6 (4.34)	4 (5.33)	2 (3.17)	**0.0174**
*Pseudomonas aeruginosa*	11 (7.97)	3 (4.00)	8 (12.69)	**<0.001**
*Klebsiela pneumoniae*	9 (6.52)	2 (2.66)	7 (11.11)	**<0.001**
Other gram-positive cocci	6 (4.34)	2 (2.66)	4 (6.34)	**0.0187**
*Enterococus species*	5 (3.62)	3 (4.00)	2 (3.17)	0.6216
*Peptostreptococcus*	3 (2.17)	2 (2.66)	1 (1.58)	0.0971
Gram-negative aerobes				
*Escherichia coli*	8 (5.79)	5 (6.66)	3 (4.76)	0.1558
*Haemophilus influenzae*	5 (3.62)	3 (4.00)	2 (3.17)	0.6882
*Acinetobacter*	6 (4.34)	2 (2.66)	4 (6.34)	**0.0311**
*Enterobacter cloacae*	4 (2.89)	2 (2.66)	2 (3.17)	0.8473
*Fusobacterium species*	1 (0.72)	0 (0)	1 (1.58)	0.1274

Qualitative variables: number (%). * Pearson Chi-square test for categorical variables. Bold *p*-values are statistically significant.

## Data Availability

The data presented in this study are available on request from the corresponding author.
